# Influence of Different Surface Modifications on the Reverse Torque Values of Abutment Screws: An In Vitro Study

**DOI:** 10.3390/jfb17090475

**Published:** 2026-09-18

**Authors:** Merve Dede, Ozgun Yusuf Ozyilmaz, Ozge Doganay Ozyilmaz, Gamze Hanci Abay

**Affiliations:** 1 Department of Prosthodontics, Faculty of Dentistry, Istanbul Galata University, 34430 Istanbul, Turkey; merve.dede@gmail.com; 2 Department of Oral and Maxillofacial Surgery, Faculty of Dentistry, Bezmialem Vakif University, 34093 Istanbul, Turkey; odoganay@bezmialem.edu.tr; 3 Private Practice, 34570 Istanbul, Turkey; dtgamzeabay@hotmail.com

**Keywords:** implant, abutment screw, torque, reverse torque, abutment screw surface

## Abstract

Evidence regarding tantalum nitride (TaN)-coated gold abutment screws is lacking, and studies on diamond-like carbon (DLC)-coated and anaerobic adhesive-coated abutment screws remain limited. This study evaluated the reverse torque values (RTVs) of abutment screws with different surface modifications. Sixty abutment screws from the same implant system were divided into five groups (*n* = 12): gold, TaN-coated gold, titanium, DLC-coated titanium, and anaerobic adhesive-coated titanium. Abutments were tightened according to the manufacturer’s recommended torque. Specimens were then subjected to cyclic loading (50 N, 1 Hz, 480,000 cycles) in a dual-axis chewing simulator, after which RTVs were evaluated. ANOVA showed a significant difference only for the uncoated gold screw group (*p* < 0.05). The lowest RTVs were observed in the gold screws. Although the differences among the modified screw groups were not statistically significant, surface-treated screws demonstrated higher and more stable RTVs than their uncoated counterparts. TaN-, DLC-, and anaerobic adhesive-treated screws exhibited higher RTVs following cyclic loading, suggesting that these surface modifications may enhance screw stability and reduce the risk of abutment screw loosening.

## 1. Introduction

Currently, implant-supported dental rehabilitation is considered the most effective treatment option for replacing missing teeth and improving patients’ quality of life [1,2,3]. Dental implant systems are generally composed of two principal components: the endosseous implant fixture, which is surgically inserted into the alveolar bone to achieve osseointegration, and the prosthetic component, which supports the definitive restoration. The prosthetic restoration is connected to the implant through an intermediate component known as the abutment, onto which a single crown, fixed partial denture, or other prosthetic restoration is fabricated. In most contemporary implant systems, the definitive crown is cemented or screw-retained on the abutment, while the abutment itself is secured to the implant fixture by an abutment screw. This screw is commonly manufactured from Ti–6Al–4V titanium alloy because of its favorable mechanical properties, corrosion resistance, and biocompatibility [4].

Despite the high long-term success rates reported, both biological and mechanical complications may occur, with prosthetic screw loosening being the most frequently described [5,6,7].

When components such as an implant and abutment, or an implant and crown, are connected by screw tightening, the interface is referred to as a screw joint. The screw is primarily subjected to two opposing forces: the clamping force, which maintains the connection, and the joint-separating force, which tends to disrupt it [8].

Factors contributing to abutment screw loosening include inadequate torque application, high occlusal loading, inaccuracies in component fabrication, and the design characteristics of both the screw and the restoration [8,9,10].

Despite continuous improvements in implant design, connection geometry, and restorative protocols, abutment screw loosening remains one of the most frequently encountered mechanical complications in implant-supported prostheses, particularly in single-unit restorations. The loss of preload generated during screw tightening may lead to micromovement at the implant–abutment interface, resulting in instability of the prosthetic assembly. Clinically, this complication may manifest as prosthesis mobility, occlusal discrepancies, patient discomfort, and impaired masticatory function. If left untreated, progressive screw loosening can ultimately result in screw fracture due to repetitive functional loading, thereby increasing the complexity of clinical management [11,12,13].

Management of fractured or severely loosened abutment screws can be particularly challenging because the fractured fragment often remains lodged within the internal connection of the osseointegrated implant. Retrieval of the broken screw frequently requires specialized instruments and meticulous clinical procedures, and successful removal cannot always be guaranteed. In addition, deformation or damage to the internal implant threads during screw loosening or retrieval may compromise the integrity of the implant–abutment connection. In such cases, preservation of the existing implant may no longer be feasible, necessitating implant removal and replacement with a new implant of larger diameter, provided that sufficient bone volume and anatomical conditions permit. These additional surgical and prosthetic interventions increase treatment time, cost, and patient morbidity, emphasizing the importance of preventive strategies aimed at improving screw stability and maintaining the mechanical integrity of the implant–abutment complex [14,15].

When screw loosening develops, the implant-supported restoration may gradually lose its mechanical stability, allowing the occurrence of micromovement at the implant–abutment interface during functional loading. Even minimal micromotion may adversely affect the integrity of the implant–prosthesis connection by increasing mechanical wear and promoting the formation of microgaps between the contacting components. These microgaps provide a favorable environment for bacterial colonization and biofilm accumulation, which may subsequently contribute to peri-implant soft tissue inflammation, unpleasant taste, halitosis, discomfort during mastication, and patient dissatisfaction [13].

Preload is defined as the tensile force generated within a screw immediately after tightening and represents one of the most important determinants of the mechanical stability of the implant–abutment complex. When tightening torque is applied, part of the torque is converted into tensile force within the screw, while the remaining energy is dissipated through friction occurring at the contacting surfaces of the screw threads and beneath the screw head. Consequently, friction and preload exhibit an inverse relationship, with lower friction generally allowing a greater proportion of the applied torque to be converted into preload. Previous investigations have demonstrated that the coefficient of friction is one of the most influential factors governing the preload achieved for a given tightening torque, emphasizing the importance of surface characteristics and material properties in maintaining screw stability over time [16]. Because preload gradually decreases as a result of settling effects and cyclic functional loading, strategies capable of minimizing friction while preserving adequate removal torque have attracted considerable interest in implant dentistry.

Recent advances in surface engineering and thin-film deposition technologies have enabled the development of highly specialized coating materials with improved mechanical and tribological properties. The availability of sophisticated deposition techniques has made it possible to apply uniform coatings with controlled thicknesses to complex implant components without substantially altering their original dimensions or mechanical fit. As a result, coating materials exhibiting superior hardness, enhanced wear resistance, improved corrosion resistance, and optimized frictional characteristics have become increasingly attractive for biomedical applications, particularly in dental implantology where long-term mechanical stability is essential [17,18]. Accordingly, several implant manufacturers have introduced dry-lubricant surface treatments, including gold coatings, diamond-like carbon (DLC) coatings, and nitride-based coatings, with the objective of reducing friction during screw tightening and thereby improving preload generation and preload maintenance [19,20].

The rationale underlying the application of diamond-like carbon (DLC) coatings is based on their exceptional combination of high hardness, excellent wear resistance, chemical inertness, and low coefficient of friction. Compared with uncoated titanium surfaces, DLC-coated screws possess a smoother and more durable surface capable of reducing frictional resistance during tightening. This reduction in friction enables a greater proportion of the applied tightening torque to be converted into preload rather than being dissipated as frictional losses. Consequently, DLC coatings have been proposed as an effective approach for improving joint stability, reducing screw loosening, and increasing the longevity of implant-supported prosthetic restorations subjected to repeated functional loading [20].

Only a limited number of experimental and laboratory studies have investigated titanium implants coated with tantalum (Ta) or tantalum nitride (TaN). The available evidence suggests that these coating materials provide several advantageous biological and mechanical characteristics, including increased resistance to microbiologically induced corrosion [21], improved mechanical strength and wear resistance, reduced bacterial adhesion to implant surfaces [22], and enhanced osseointegration through improved cellular responses [23]. These favorable properties indicate that Ta- and TaN-based coatings may represent promising alternatives for enhancing the long-term clinical performance of implant components. Nevertheless, based on the available literature, no previous studies have specifically evaluated the application of TaN coatings to titanium or gold abutment screws or investigated their influence on reverse torque values following cyclic mechanical loading. Therefore, the potential mechanical advantages of this surface modification remain largely unexplored in implant prosthodontics.

Therefore, the present study was designed to address this gap by comparing the reverse torque values of TaN-coated gold abutment screws with those of conventional titanium, gold, DLC-coated titanium, and anaerobic adhesive-treated titanium screws under standardized cyclic loading conditions.

More recently, anaerobic adhesives have emerged as another promising strategy for improving the mechanical stability of implant-abutment screw joints. These single-component resin-based materials have been widely used in industrial and mechanical engineering applications for many years because of their ability to polymerize in the absence of oxygen after confinement between metallic surfaces. Once polymerized, the adhesive fills the microscopic spaces between the screw threads, thereby increasing thread engagement, improving sealing of the screw joint, and reducing the likelihood of spontaneous screw loosening during functional loading. Several investigations have reported encouraging mechanical outcomes following the application of anaerobic adhesives, particularly with respect to increasing reverse torque values and improving preload maintenance across different implant connection geometries [24,25,26]. However, only a limited number of studies have evaluated their performance specifically at the implant–abutment interface, and most of these investigations have been performed under static loading conditions rather than dynamic cyclic loading, which more closely resembles the clinical environment [26,27,28]. In the present study, LOCTITE 243 (Henkel AG & Co. KGaA, Düsseldorf, Germany) was therefore incorporated into the experimental design to evaluate its potential influence on reverse torque values following simulated mastication.

Accordingly, the objective of the present in vitro investigation was to compare the reverse torque values of different implant abutment screw materials and surface treatments, including conventional titanium screws, gold screws, TaN-coated gold screws, DLC-coated titanium screws, and titanium screws treated with an anaerobic adhesive, following cyclic mechanical loading in a chewing simulator. The null hypothesis was that neither the application of anaerobic adhesive nor the different surface coating materials would significantly influence the reverse torque values after cyclic loading when compared with their corresponding uncoated abutment screws.

## 2. Materials and Methods

An effect size of 0.40 was determined based on previous studies investigating surface treatments of implant abutments [19,20,24,25,26]. With a significance level of α = 0.05 and a statistical power of 0.80, the minimum required sample size was calculated as *n* = 6 per subgroup; this number was increased to *n* = 12 to improve the robustness of the findings and reduce variability among specimens.

**Figure 1:**
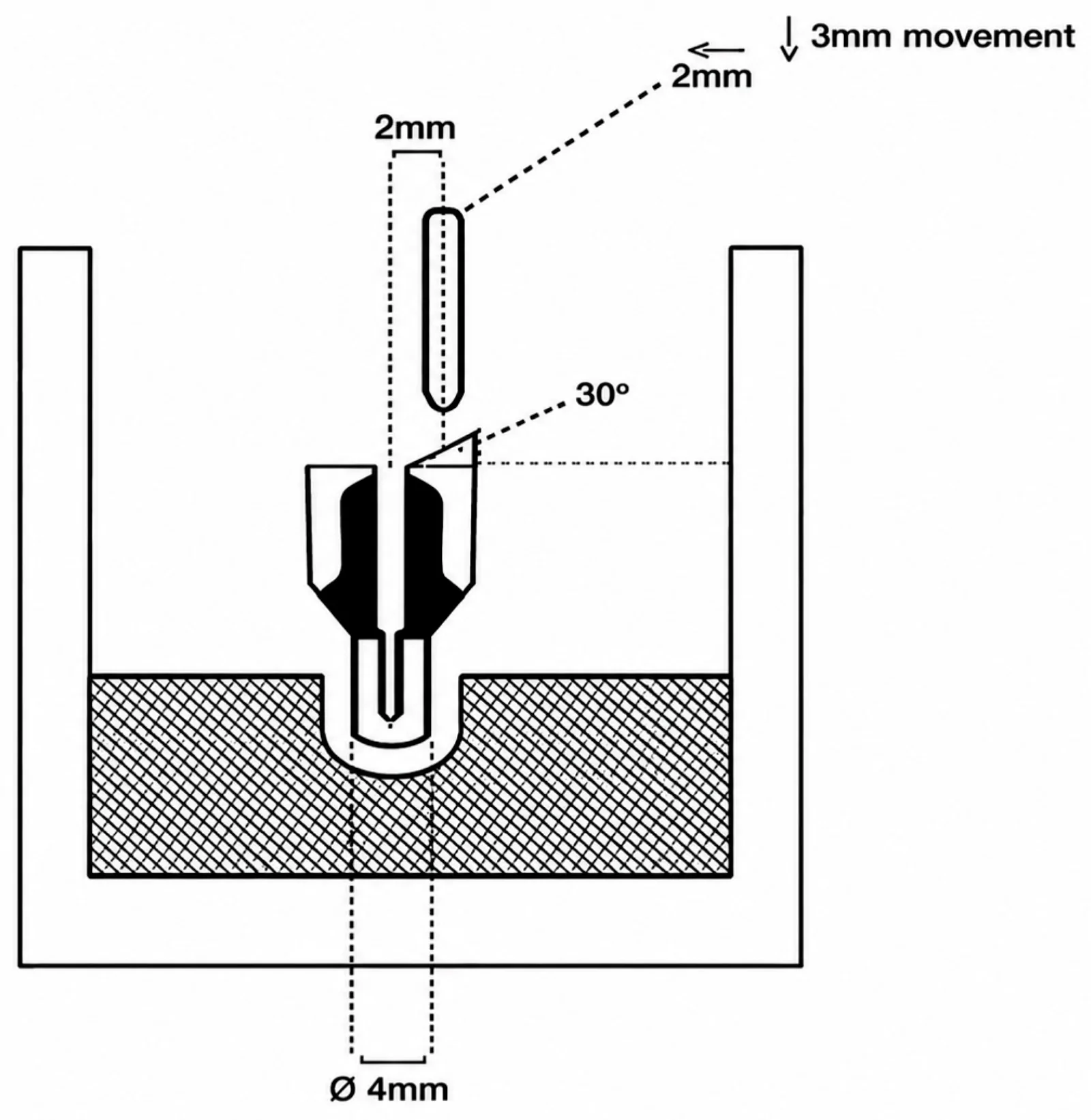
View of the chewing simulator in which the dynamic loadings were performed.

A total of 60 internal hexagon implants (4 mm × 10 mm, non-platform switched; Biomet 3i, Barcelona, Spain) together with their corresponding straight abutments were included in this in vitro investigation. Each experimental specimen consisted of a single implant–abutment assembly. To simulate the biomechanical characteristics of the implant–bone complex under clinical conditions, all implants were embedded vertically in epoxy resin (EpoFix Kit; Struers, Ballerup, Denmark) possessing an elastic modulus comparable to that of human cortical bone. Straight abutments (4.1 mm in diameter, 5 mm in height, and a 2 mm transmucosal collar) were connected to the implants using abutment screws. The specimens were embedded in epoxy resin, which also served to stabilize them within custom-designed holders specifically fabricated for the chewing simulator. The implant platform was positioned at the resin surface and remained fully exposed; therefore, no additional resin coverage was present above the implant platform. The implants were embedded vertically to provide standardized alignment during testing. Full-metal crowns were fabricated using a cobalt–chromium (Co-Cr) alloy and the crowns with standardized 30° cusp inclinations were fabricated for all abutments and luted with a dual-polymerizing resin cement (RelyX U200; 3M ESPE, St. Paul, MN, USA) (Figure 1). The crowns were designed with screw access holes, which were sealed with Teflon tape before cementation. After cementation, the crowns were torqued to the manufacturer’s recommended tightening torque.

The specimens were randomly allocated into five experimental groups (*n* = 12 per group) according to the type and surface characteristics of the abutment screw. A total of 60 abutment screws from the same implant system were evaluated, comprising Certain Gold-Tite Hexed gold screws, tantalum nitride (TaN)-coated gold screws, Certain titanium hexed screws, diamond-like carbon (DLC)-coated titanium screws, and titanium screws treated with an anaerobic adhesive.

The gold abutment screws were coated with tantalum nitride (TaN) using the direct current (DC) magnetron sputtering technique. Before deposition, the coating chamber was evacuated to a base pressure of 1 × 10^−3^ Pa to eliminate residual gases and ensure optimal coating conditions. Initially, a tantalum (Ta) interlayer was deposited for 20 min under an argon atmosphere at a working pressure of 1 Pa to improve coating adhesion. Subsequently, the TaN coating was deposited for 60 min using an argon/nitrogen gas mixture (4:1) at the same pressure, producing a homogeneous coating with a final thickness of approximately 1.2 μm.

For the diamond-like carbon (DLC)-coated titanium screws, the coating chamber was first evacuated to a base pressure of 7.0 × 10^−4^ Pa. A chromium (Cr) interlayer was then deposited for 5 min under argon gas at a pressure of 0.6 Pa using the DC magnetron sputtering technique to enhance the adhesion between the substrate and the DLC layer. Subsequently, the DLC coating was applied by radio-frequency (RF) magnetron discharge using methane (CH_4_) gas at a pressure of 2 Pa for 150 min, resulting in a uniform coating with an average thickness of 1.3 μm.

**Figure 2:**
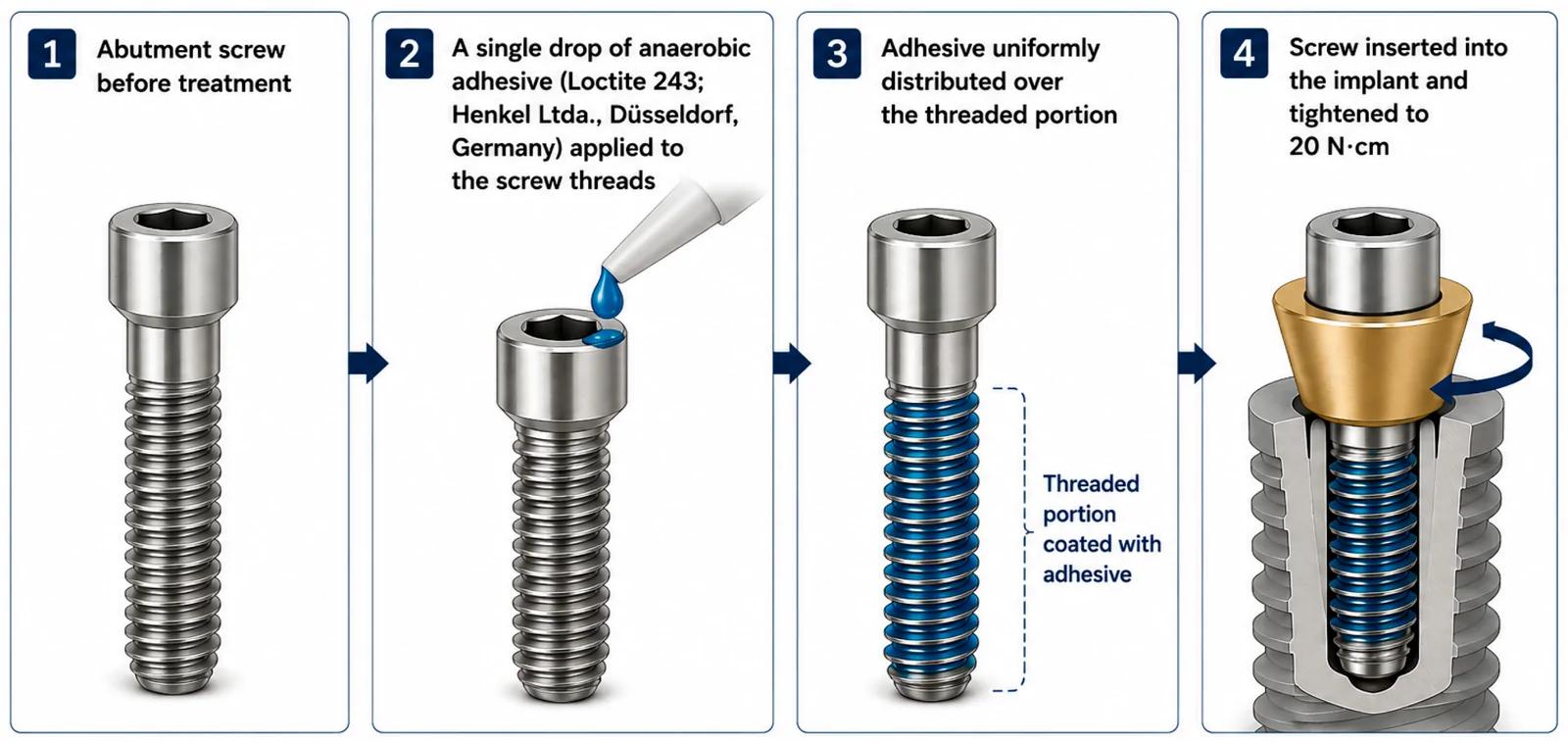
Schematic illustration of the standardized application procedure of the anaerobic adhesive. (**1**) Abutment screw before treatment. (**2**) A single drop of adhesive (Loctite 243; Henkel AG & Co. KGaA, Düsseldorf, Germany) applied to screw threads. (**3**) Uniform distribution of the adhesive over the threaded portion. (**4**) Screw insertion into the implant and tightening to the manufacturer’s recommended torque value (20 N·cm). Schematic illustration created by artificial intelligence.

For the anaerobic adhesive group, a single drop of a commercially available anaerobic adhesive (Loctite 243; Henkel AG & Co. KGaA, Düsseldorf, Germany) (Figure 2) was carefully applied to the threads of each titanium abutment screw before insertion.

**Figure 3:**
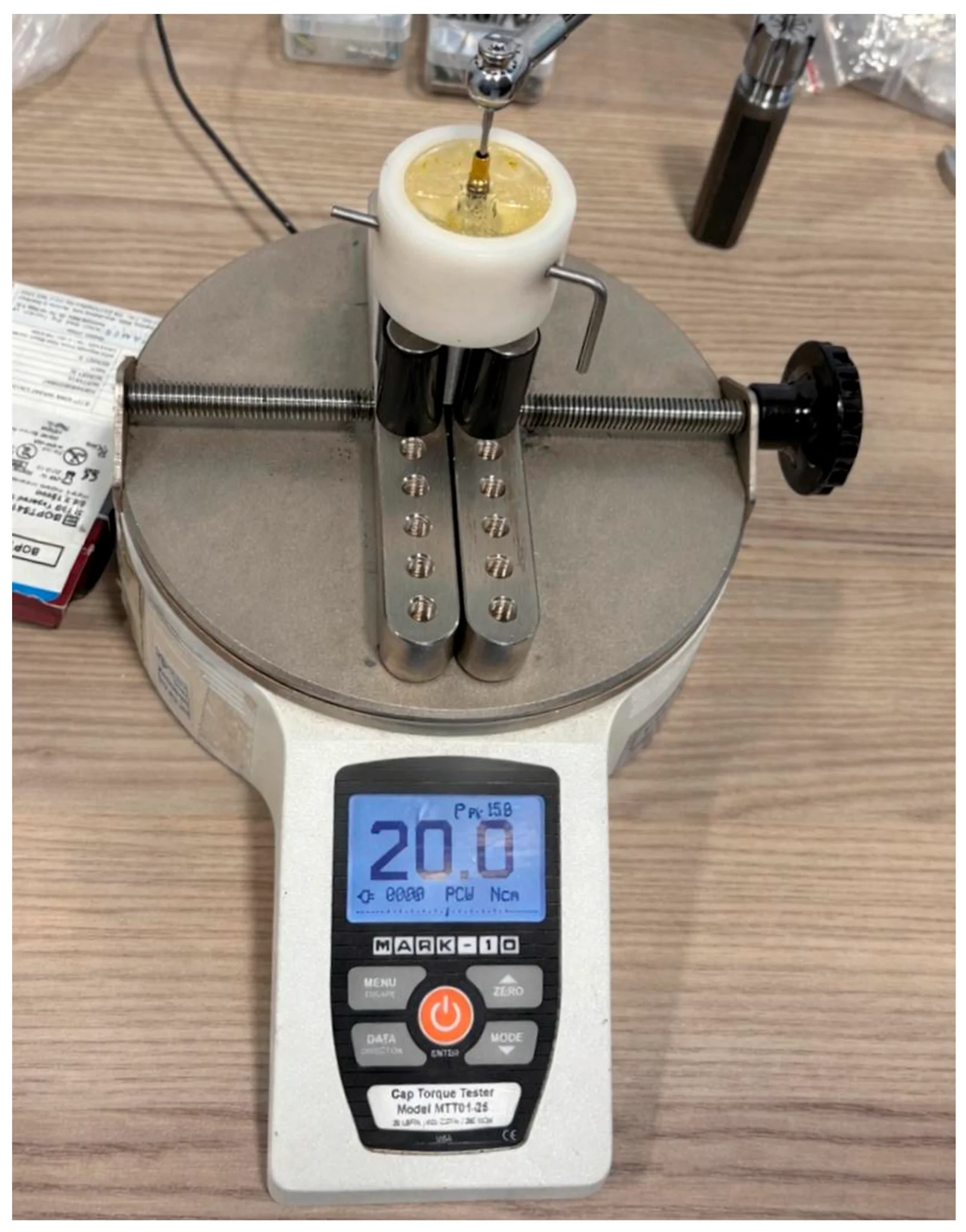
Digital torque meter. Every abutment screw to the manufacturer’s recommended tightening torque of 20 N·cm and the reverse torque values (RTVs) were measured after they were removed from the chewing simulator.

After completion of the surface treatments of the abutment screws, every abutment screw to the manufacturer’s recommended tightening torque of 20 N·cm using a calibrated digital torque meter (Cap Torque Tester MTT01-25, MARK-10 Corporation, Copiague, NY, USA) (Figure 3). Following a waiting period of 10 min, the same operator retightened every abutment screw to minimize the influence of the settling effect and maximize torque stability. The torque meter has a torque capacity of 290 N·cm, a resolution of 0.2 N·cm, and an accuracy of ±0.3% of full scale. The device was calibrated according to the manufacturer’s recommendations.

**Figure 4:**
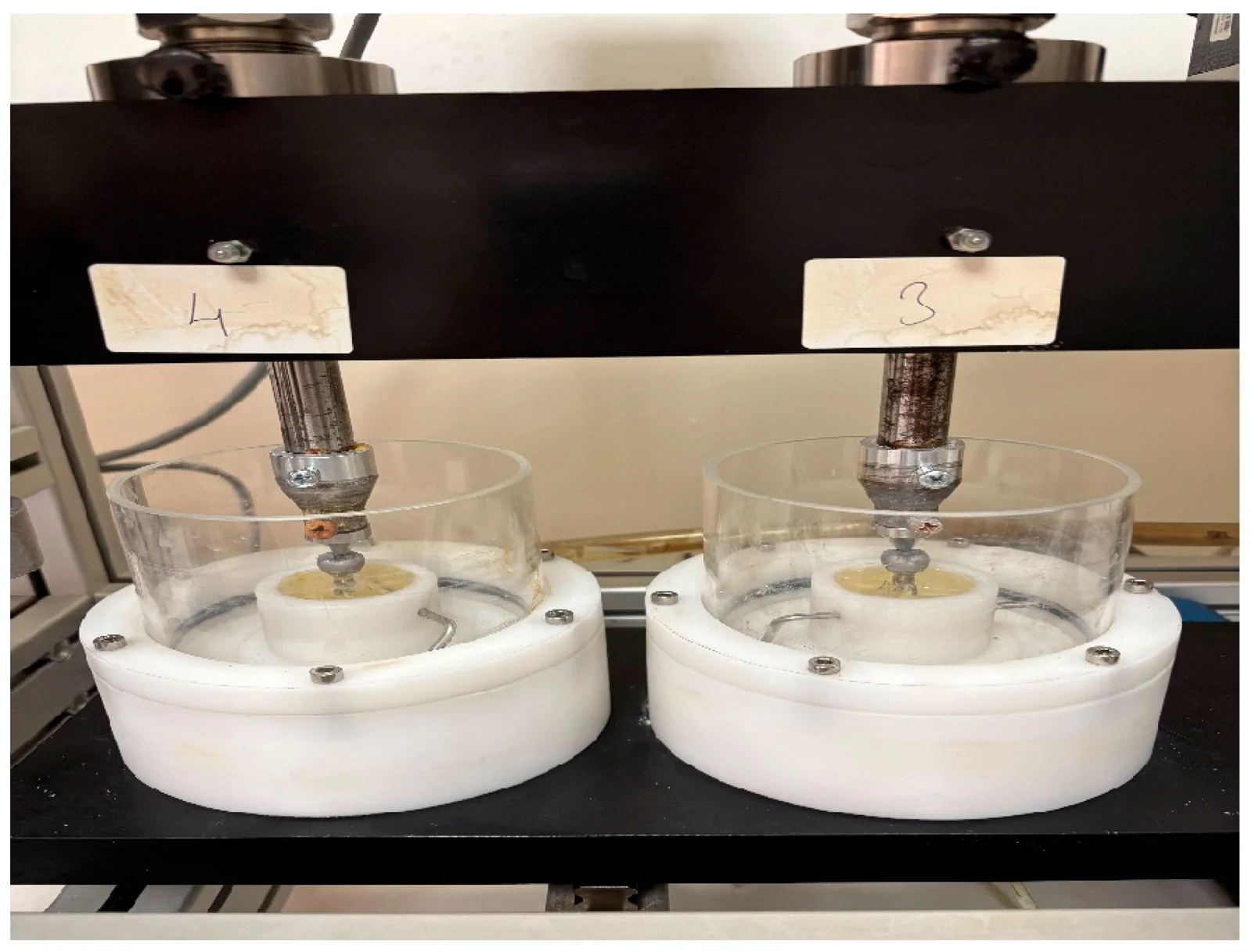
Chewing simulator. A cyclic load of 50 N was applied at a frequency of 1 Hz for a total of 480,000 loading cycles, corresponding to approximately two years of clinical function and remaining within the accepted physiological range of occlusal forces.

Following assembly, the specimens were subjected to dynamic mechanical fatigue using a dual-axis chewing simulator (CS-4.4 Chewing Simulator, SD Mechatronik GmbH, Feldkirchen-Westerham, Germany) (Figure 4), which allowed simultaneous testing of four specimens. A cyclic load of 50 N was applied at a frequency of 1 Hz for a total of 480,000 loading cycles, corresponding to approximately two years of clinical function and remaining within the accepted physiological range of occlusal forces [29,30,31]. Mechanical loading was performed under dry conditions without the use of any liquid medium. During each loading cycle, a rounded stainless-steel stylus initially contacted the occlusal surface at a point located 2 mm away from the center of the crown, moved vertically downward by 3 mm, and then slid horizontally for 2 mm toward the center of the restoration, thereby simulating the functional movements occurring during mastication. Upon completion of cyclic loading, all specimens were removed from the chewing simulator, and the reverse torque values (RTVs) were measured by the same operator using the digital torque meter.

The reverse torque value (RTV) was defined as the maximum torque required to initiate screw loosening after cyclic loading. Each screw was unscrewed using the digital torque meter, and the peak value displayed at the onset of screw rotation was recorded as the RTV.

### Statistical Analysis

All statistical analyses were performed using IBM SPSS Statistics version 28 (IBM Corp., Armonk, NY, USA). The distribution of the data was assessed using the Shapiro–Wilk test, while the homogeneity of variances was evaluated using Levene’s test. Because the data demonstrated normal distribution and homogeneity of variance, comparisons among the five experimental groups were carried out using one-way analysis of variance (ANOVA). When statistically significant differences were identified, Tukey’s honestly significant difference (HSD) post hoc test was applied to determine which groups were responsible for the observed differences. Statistical significance was established at *p* < 0.05.

## 3. Results

Descriptives of reverse torque values (N·cm) of materials evaluated together with their corresponding standard deviations for the five experimental groups following cyclic loading are presented in Table 1, Table 2 and Table 3.

Tukey’s post hoc test demonstrated that a statistically significant difference was observed only for the uncoated gold screw group, which exhibited significantly lower RTVs than the remaining experimental groups (*p* < 0.05). No statistically significant differences were identified among the titanium screws, DLC-coated titanium screws, anaerobic adhesive-coated titanium screws, and TaN-coated gold screws.

The mean RTVs recorded for the titanium, DLC-coated titanium, anaerobic adhesive-coated titanium, and TaN-coated gold groups were 16.12 N·cm, 16.44 N·cm, 16.55 N·cm, and 16.36 N·cm, respectively. Although the highest mean RTV was observed in the titanium screws treated with the anaerobic adhesive, this value did not differ significantly from those obtained for the titanium, DLC-coated titanium, or TaN-coated gold groups.

The lowest mean RTV was recorded in the uncoated gold screw group (12.42 N·cm), and this reduction was found to be statistically significant compared with the other experimental groups.

Although the increases in RTVs produced by the different surface modification techniques did not reach statistical significance, both the titanium-based and gold-based surface treatments consistently resulted in higher reverse torque values than those observed in their corresponding uncoated control groups, suggesting an overall trend toward reduced screw loosening following cyclic mechanical loading.

## 4. Discussion

The present study was designed to investigate the influence of different surface modifications applied to abutment screws used in single implant-supported restorations and to evaluate their effects on reverse torque values (RTVs) following cyclic mechanical loading in a chewing simulator. The results demonstrated that, with the exception of the uncoated gold screw group, no statistically significant differences were observed among the experimental groups. Nevertheless, the gold screw group exhibited significantly lower RTVs than the other groups, indicating a greater loss of preload after mechanical loading. The anaerobic adhesive group showed the numerically highest mean RTV; however, it did not differ significantly from the titanium, DLC-coated titanium, or TaN-coated gold groups.

Based on these findings, the null hypothesis that surface modification of Universal Abutment screws would have no influence on reverse torque values following cyclic loading was not uniformly supported or rejected. Although a statistically significant overall difference was observed among the experimental groups, the pairwise comparisons indicated that this difference was primarily attributable to the significantly lower RTVs of the uncoated gold screw group. No statistically significant differences were observed among the uncoated titanium, DLC-coated titanium, anaerobic adhesive-treated titanium, and TaN-coated gold screw groups. Thus, the present findings suggest that the observed overall difference was driven primarily by the lower RTVs of the uncoated gold screws rather than by consistent differences among the surface-modified screw groups.

Reverse torque values were measured after cyclic mechanical loading and were used to evaluate the mechanical behavior of the abutment screws following loading. Because initial reverse torque values were not measured immediately after screw tightening, the post-loading RTVs cannot be directly compared with baseline RTVs or interpreted as a direct measure of torque loss from the initial condition. Tightening torque represents the torque applied during screw installation, whereas reverse torque represents the torque required to remove the screw after loading; therefore, these two parameters should not be considered equivalent. The observed differences in post-loading RTVs among the groups may reflect the effects of settling and cyclic mechanical loading on the implant–abutment connection. Previous investigations have similarly used RTV as an indirect parameter for evaluating screw stability following mechanical loading [32,33,34]. The observed post-loading RTVs may be influenced by the well-established settling effect, in which microscopic surface irregularities at the contacting interfaces become flattened after tightening, resulting in a partial loss of preload. Furthermore, cyclic mechanical loading produces repeated micromovements at the implant–abutment interface, which progressively reduce frictional resistance between the contacting surfaces and facilitate gradual screw loosening during functional loading.

Single implant-supported restorations were selected as the experimental model because abutment screw loosening is reported considerably more frequently in single-unit restorations than in other prosthetic configurations. Despite substantial improvements in implant surface characteristics, connection geometries, and restorative protocols, screw loosening continues to represent the most common mechanical complication associated with single implant-supported prostheses. Although this complication can also occur in multiple implant-supported fixed prostheses, its reported incidence is considerably lower than that observed in single-unit restorations [35,36]. Consequently, evaluating methods capable of improving screw stability in single implants remains highly relevant from a clinical perspective.

In the present study, all abutment screws were retightened 10 min after the torque application to prevent screw loosening associated with the settling effect. This procedure was standardized for all experimental groups to ensure comparable testing conditions. A similar protocol was recommended by Siamos et al. [32], who investigated the influence of retightening on premachined abutments and concluded that routinely retorquing abutment screws approximately 10 min after the tightening helps establish a more stable implant–abutment connection by compensating.

The performance of the DLC-coated titanium screws observed in the present study is consistent with previously published investigations evaluating similar surface modifications. Earlier studies comparing conventional titanium screws with DLC-coated titanium screws reported higher reverse torque values for the coated screws, indicating superior preload maintenance following cyclic loading, findings that closely resemble those obtained in the current investigation [29]. Additional studies have likewise demonstrated that DLC-coated titanium screws generally exhibit improved RTVs compared with uncoated titanium screws [16,37]. These observations have been explained by the ability of DLC coatings to reduce friction during screw tightening, thereby improving the mechanical integrity of the implant–abutment connection. This mechanism has been proposed to contribute to higher reverse torque values after cyclic loading [38]. However, preload was not measured in the present study; therefore, this explanation should be interpreted as a possible mechanism reported in the literature rather than a finding of the present investigation.

Nevertheless, the literature does not report entirely consistent findings regarding DLC coatings. In contrast to the results of the present study, certain investigations have reported lower reverse torque values for DLC-coated titanium screws following cyclic loading [37]. These contradictory findings have been attributed to the possibility that, although DLC coatings have been reported to reduce friction during screw tightening and may influence preload generation, the same reduction in friction may also decrease the resistance encountered during screw removal. Consequently, the lower frictional resistance during unscrewing may lead to reduced reverse torque values despite the presence of a relatively high torque values [39].

Compared with DLC coatings, considerably fewer studies have evaluated titanium implants coated with tantalum (Ta) or tantalum nitride (TaN). The available literature indicates that these surface modifications provide several favorable biological and mechanical properties, including increased resistance to microbiologically induced corrosion [21], enhanced mechanical strength and wear resistance, reduced bacterial adhesion [22], and improved osseointegration of dental implants [23]. However, based on the available literature, no previous investigation has evaluated TaN-coated gold abutment screws or examined their influence on reverse torque values in implant dentistry. Existing studies evaluating various coated implant screws have consistently reported improvements in wear resistance associated with surface coatings [17,18,19,20]. These previously reported findings support the results obtained in the present investigation, in which TaN-coated gold screws demonstrated higher reverse torque values than uncoated gold screws following cyclic mechanical loading.

The results obtained for the anaerobic adhesive group are also in agreement with previous investigations evaluating similar adhesive materials. Studies in which anaerobic adhesives comparable to the material used in the present investigation were applied to implant abutment screws reported that substantially greater torque was required to remove the screws after loading, suggesting that the theoretical probability of screw loosening may be reduced through the use of such materials [24,26,27]. According to the manufacturer’s technical documentation, the anaerobic adhesive is specifically intended to provide both mechanical retention and sealing of threaded components while maintaining the possibility of routine screw retrieval using conventional hand instruments [40]. According to the manufacturer’s technical documentation, the adhesive is intended to maintain the retrievability of threaded components. However, retrievability was not specifically evaluated, only reverse torque values were evaluated in the present study.

However, not all previously published studies have reported beneficial effects associated with anaerobic adhesives. In contrast to present study, one investigation demonstrated lower reverse torque values in the anaerobic adhesive group and suggested that this unexpected outcome might have resulted from polymerization shrinkage of the adhesive material following application [31]. Such inconsistencies indicate that the mechanical behavior of anaerobic adhesives may be influenced by several variables, including adhesive formulation, application technique, testing methodology, and loading conditions.

The authors attributed these findings primarily to the relatively low elastic modulus of silver (E = 69 GPa) compared with titanium (E = 110 GPa). Because of its lower stiffness and greater capacity for elastic deformation, silver allows the screw threads to adapt more intimately to the internal implant threads, thereby increasing the contact area at the screw–thread interface. This enlarged contact interface increases frictional resistance during functional loading and contributes to a more stable and mechanically secure implant–abutment connection [41]. Similarly, Bulaqi et al. [42] reported that the elastic modulus of gold abutments was approximately 136 GPa, a value considerably higher than that of titanium. The higher stiffness of gold may reduce its ability to conform to the internal implant threads during tightening, potentially limiting the extent of thread engagement and reducing frictional resistance. Consequently, gold screws would be expected to exhibit lower reverse torque values than titanium screws, an observation that is consistent with the findings obtained in the present investigation.

Overall, the findings of the present study demonstrated that the tested surface modifications produced higher reverse torque values than the uncoated gold screws after cyclic loading. Although reverse torque values are frequently used as an indirect mechanical parameter in implant studies, they should not be interpreted as direct measures of preload, sealing ability, microbial leakage, or long-term clinical performance. Further investigations evaluating these outcomes directly are required to determine whether the observed differences in reverse torque values have clinical significance. Furthermore, it should be recognized that screw loosening frequently represents one of the earliest clinical signs of biomechanical instability, often presenting as component misfit, occlusal discrepancies, or evidence of parafunctional loading before more severe mechanical complications, including screw fracture or prosthetic failure, become clinically evident [43,44].

Several limitations of the present study should be acknowledged. First, post-fatigue characterization of the coatings was not performed. Surface morphology, elemental composition, coating thickness, surface roughness, adhesion, and coating integrity were not evaluated before or after cyclic mechanical loading. Therefore, potential changes in the coating characteristics and their relationship with the observed reverse torque values could not be directly assessed. Future studies should incorporate comprehensive coating characterization, including scanning electron microscopy (SEM), energy-dispersive spectroscopy (EDS), coating thickness verification, surface roughness measurements, and coating adhesion/integrity testing.

Second, the present study was conducted under in vitro conditions using a dry environment and a standardized cyclic loading protocol. Therefore, the findings cannot be directly extrapolated to clinical conditions. Further in situ and in vivo studies are needed to evaluate the effects of the tested treatments under clinically relevant conditions, including exposure to oral fluids, thermal changes, and biological factors, as well as to assess their long-term biocompatibility and cytotoxicity.

Third, only one implant system and a single-unit implant-supported prosthetic design were evaluated. Because implant connection geometry, component design, and prosthetic configuration may influence reverse torque values, future studies should investigate the tested surface treatments using different implant systems and prosthetic designs to determine whether the present findings are consistent across different configurations.

## 5. Conclusions

The uncoated gold screw group exhibited significantly lower post-cyclic loading reverse torque values (RTVs) than the other experimental groups (*p* < 0.05). Although numerically higher mean RTVs were observed for the TaN-coated gold, DLC-coated titanium, and anaerobic adhesive groups, no statistically significant differences were detected among these groups. The highest mean RTV was observed in the anaerobic adhesive group; however, this difference was not statistically significant. These findings indicate that, under the dry in vitro conditions and cyclic loading protocol used in the present study, the tested surface treatments and anaerobic adhesive application did not result in statistically significant differences in RTV among the non-gold groups. Further studies under clinically relevant conditions, including liquid medium, thermal cycling, and longer-term mechanical loading, are warranted to further evaluate the effects of these treatments on RTV and their potential clinical relevance.

## Figures and Tables

**Table 1:** Results of the one-way ANOVA test.

	Sum of Squares	Df	Mean Square	F	Sig
Between Groups	150.959	4	37.740	90.935	<0.001
Within Groups	22.826	55	0.415		
Total	173.785	59			

**Table 2:** Descriptives of tork values (N) of materials evaluated.

Materials	Mean	Std. Deviation	Minimum	Maximum
Ti	16.12 ^a^	0.72	14.80	17.06
DLC	16.44 ^a^	0.65	15.20	17.40
Yapışkan	16.55 ^a^	0.64	15.52	17.76
Altın	12.42 ^b^	0.34	11.80	12.80
TaN	16.36 ^a^	0.76	15.00	17.60
+ p	*			

+ One-way ANOVA-Tukey HSD. Lowercase letters in-group indicate statistical difference between groups. * *p* < 0.05.

**Table 3:** Multiple comparisons of groups with Tukey HSD post hoc test.

		Mean Difference	Std Error	Sig.	Lower Bond	Upper Bond
Ti	DLC	−0.32083	0.26300	0.740	−1.0626	0.4209
Ti	Anaerobic adhesive	−0.43500	0.26300	0.470	−1.1768	0.3068
Ti	Gold	3.7008	0.26300	<0.001	2.9591	4.4426
Ti	TaN	−0.23833	0.26300	0.893	−0.9801	0.5034
DLC	Anaerobic adhesive	−0.11417	0.26300	0.992	−0.8559	0.6276
DLC	Gold	4.0216	0.26300	<0.001	3.2799	4.7634
DLC	TaN	0.08250	0.26300	0.998	−0.6593	0.8243
Anaerobic adhesive	Gold	4.1358	0.26300	<0.001	3.3941	4.8776
Anaerobic adhesive	TaN	0.19667	0.26300	0.944	−0.5451	0.9384
TaN	Gold	3.9391	0.26300	<0.001	3.1974	4.6809

## Data Availability

The data that support the findings of this study are available from the corresponding author upon reasonable request.

## References

[1] Ali Z., Baker S.R., Shahrbaf S., Martin N., Vettore M.V. (2019). Oral health-related quality of life after prosthodontic treatment for patients with partial edentulism: A systematic review and meta-analysis. J. Prosthet. Dent..

[2] Reissmann D.R., Dard M., Lamprecht R., Struppek J., Heydecke G. (2017). Oral health-related quality of life in subjects with implant-supported prostheses: A systematic review. J. Dent..

[3] Silva A.S., Martins D., de Sá J., Mendes J.M. (2021). Clinical evaluation of the implant survival rate in patients subjected to immediate implant loading protocols. Dent. Med. Probl..

[4] Branemark P.I. (1983). Osseointegration and its experimental background. J. Prosthet. Dent..

[5] Pjetursson B.E., Thoma D., Jung R., Zwahlen M., Zembic A. (2012). A systematic review of the survival and complication rates of implant-supported fixed dental prostheses (FDPs) after a mean observation period of at least 5 years. Clin. Oral Implant. Res..

[6] Zembic A., Kim S., Zwahlen M., Kelly J.R. (2014). Systematic Review of the Survival Rate and Incidence of Biologic, Technical, and Esthetic Complications of Single Implant Abutments Supporting Fixed Prostheses. Int. J. Oral Maxillofac. Implant..

[7] Jung R.E., Pjetursson B.E., Glauser R., Zembic A., Zwahlen M., Lang N.P. (2008). A systematic review of the 5-year survival and complication rates of implant-supported single crowns. Clin. Oral Implant. Res..

[8] McGlumphy E.A., Mendel D.A., Holloway J.A. (1998). Implant screw mechanics. Dent. Clin. N. Am..

[9] Jomeus L., Eng M., Jemt T., Carlsson L., Eng E. (1992). Loads and Designs of Screw Joints for Single Crowns Supported by Osseointegrated Implants. Int. J. Oral Maxillofac. Implant..

[10] Binon P., Sutter F., Beaty K., Brunski J., Gulbransen H., Weiner R. (1994). The role of screws in implant systems. Int. J. Oral Maxillofac. Implant..

[11] Lee K.Y., Shin K.S., Jung J.H., Cho H.W., Kwon K.H., Kim Y.L. (2020). Clinical study on screw loosening in dental implant prostheses: A 6-year retrospective study. J. Korean Assoc. Oral Maxillofac. Surg..

[12] Calderon P.S., Dantas P.M.C., Montenegro S.C.L., Carreiro A.F.P., Oliveira Â.G.R.C., Dantas E.M., Gurgel B.C.V. (2014). Technical complications with implant-supported dental prostheses. J. Oral Sci..

[13] Pardal-Peláez B., Montero J. (2017). Preload loss of abutment screws after dynamic fatigue in single implant-supported restorations. A systematic review. J. Clin. Exp. Dent..

[14] Al Jabbari Y.S., Fournelle R., Ziebert G., Toth J., Iacopino A.M. (2008). Mechanical behavior and failure analysis of prosthetic retaining screws after long-term use in vivo. Part 1: Characterization of adhesive wear and structure of retaining screws. J. Prosthodont..

[15] Tsuge T., Hagiwara Y. (2009). Influence of lateral-oblique cyclic loading on abutment screw loosening of internal and external hexagon implants. Dent. Mater. J..

[16] De Moura M.B., Rodrigues R.B., Pinto L.M., De Araújo C.A., Novais V.R., Júnior P.C.S. (2017). Influence of screw surface treatment on retention of implant-supported fixed partial dentures. J. Oral Implantol..

[17] Santecchia E., Hamouda A.M.S., Musharavati F., Zalnezhad E., Cabibbo M., Spigarelli S. (2015). Wear resistance investigation of titanium nitride-based coatings. Ceram. Int..

[18] Tkadletz M., Schalk N., Daniel R., Keckes J., Czettl C., Mitterer C. (2016). Advanced characterization methods for wear resistant hard coatings: A review on recent progress. Surf. Coat. Technol..

[19] Byrne D., Jacobs S., O’Connell B., Houston F., Claffey N. (2006). Preloads generated with repeated tightening in three types of screws used in dental implant assemblies. J. Prosthodont..

[20] Kim S.K., Lee J.B., Koak J.Y., Heo S.J., Lee K.R., Cho L.R., Lee S.S. (2005). An abutment screw loosening study of a Diamond Like Carbon-coated CP titanium implant. J. Oral Rehabil..

[21] Zhang Y., Zheng Y., Li Y., Wang L., Bai Y., Zhao Q., Xiong X., Cheng Y., Tang Z., Deng Y. (2015). Tantalum nitride-decorated titanium with enhanced resistance to microbiologically induced corrosion and mechanical property for dental application. PLoS ONE.

[22] Gao J., Wang M. (2021). Atomic layer deposition of TaN thin film on Ti–6Al–4V dental implant for enhanced anti-corrosion and anti-bacterial properties. J. Phys. Chem. Solids.

[23] Cui J., Zhang S., Huang M., Mu X., Hei J., Yau V., He H. (2023). Micro-nano porous structured tantalum-coated dental implants promote osteogenic activity in vitro and enhance osseointegration in vivo. J. Biomed. Mater. Res. Part A.

[24] Seloto C., Sahyon H., dos Santos P., Delben J., Assunção W. (2018). Efficacy of Sealing Agents on Preload Maintenance of Screw-Retained Implant-Supported Prostheses. Int. J. Oral Maxillofac. Implant..

[25] Ryu S.B., Heo S.J., Koak J.Y., Kim S.K. (2020). Effects of anaerobic sealing agents on preload maintenance ofscrew-retained implant prosthesis with surface of screws. J. Korean Acad. Prosthodont..

[26] Lyra F.T.M., Mares C.A., Ana Flor S., Dias D.R., Lages F.S. (2023). Anaerobic adhesives effect on counter-torque of abutment screws on implants with external hexagon and conical connections: An in vitro study. Clin. Implant Dent. Relat. Res..

[27] Nascimento L.R.M., da Silva Resende C., Ana Flor S., Francescato O., Dias D.R., Lages F.S. (2025). Anaerobic Adhesive Effect on the Counter-Torque of Zirconia Implant Abutment Screws: In Vitro Study. Clin. Implant Dent. Relat. Res..

[28] Seloto C.B., Strazzi-Sahyon H.B., Dos Santos P.H., Assunção W.G. (2021). Performance of different abutment/implant joints as a result of a sealing agent. J. Prosthodont. Res..

[29] Colpak E., Gumus H. (2020). Effect of Surface Modifications of Abutment Screws on Reverse Torque Values: An In Vitro Study. Int. J. Prosthodont..

[30] Steinebrunner L., Wolfart S., Ludwig K., Kern M. (2008). Implant-abutment interface design affects fatigue and fracture strength of implants. Clin. Oral Implant. Res..

[31] Ozdiler A., Dayan S.C., Gencel B., Ozkol G.I. (2021). Reverse torque values of abutment screws after dynamic loading: Effects of sealant agents and the taper of conical connections. J. Oral Implantol..

[32] Siamos G., Winkler S., Boberick K.G. (2002). Relationship between implant preload and screw loosening on implant-supported prostheses. J. Oral Implantol..

[33] Cantwell A., Hobkirk J.A. (2004). Preload loss in gold prosthesis-retaining screws as a function of time. Int. J. Oral Maxillofac. Implant..

[34] Jaarda M.J., Razzoog M.E., Gratton D.G. (1993). Providing optimum torque to implant prostheses: A pilot study. Implant Dent..

[35] Arshad M., Shirani G., Refoua S., Yeganeh M.R. (2017). Comparative study of abutment screw loosening with or without adhesive material. J. Adv. Prosthodont..

[36] Sailer I., Karasan D., Todorovic A., Ligoutsikou M., Pjetursson B.E. (2022). Prosthetic failures in dental implant therapy. Periodontol. 2000.

[37] Bacchi A., Regalin A., Brilhante Bhering C.L., Alessandretti R., Spazzin A.O. (2015). Loosening torque of Universal Abutment screws after cyclic loading: Influence of tightening technique and screw coating. J. Adv. Prosthodont..

[38] Basílio M.A., Butignon L.E., Arioli F.J.N. (2012). Effectiveness of screw surface coating on the stability of zirconia abutments after cyclic loading. Int. J. Oral Maxillofac. Implant..

[39] Park J.K., Choi J.U., Jeon Y.C., Choi K.S., Jeong C.M. (2010). Effects of abutment screw coating on implant preload. J. Prosthodont..

[40] Henkel (2020). Loctite 243 Technical Data Sheet.

[41] Velasco-Ortega E., Jiménez-Guerra A., Ortiz-Garcia I., Nuñez-Márquez E., Moreno-Muñoz J., Gil J., Delgado L.M., Rondón-Romero J.L., Monsalve-Guil L. (2023). Silver coating on dental implant-abutment connection screws as potential strategy to prevent loosening and minimizing bacteria adhesion. Front. Bioeng. Biotechnol..

[42] Bulaqi H.A., Barzegar A., Paknejad M., Safari H. (2019). Assessment of preload, remaining torque, and removal torque in abutment screws under different frictional conditions: A finite element analysis. J. Prosthet. Dent..

[43] Assunção W.G., Barão V.A.R., Delben J.A., Gomes É.A., Garcia I.R. (2011). Effect of unilateral misfit on preload of retention screws of implant-supported prostheses submitted to mechanical cycling. J. Prosthodont. Res..

[44] de Assis Vianna C., Delben J.A., Barao V.A.R., Ferreira M.B., dos Santos P.H., Assuncao W.G. (2013). Torque stability of different abutment screws submitted to mechanical cycling. Int. J. Oral Maxillofac. Implant..

